# Triphenylamine, Carbazole or Tetraphenylethylene-Functionalized Benzothiadiazole Derivatives: Aggregation-Induced Emission (AIE), Solvatochromic and Different Mechanoresponsive Fluorescence Characteristics

**DOI:** 10.3390/molecules27154740

**Published:** 2022-07-25

**Authors:** Yue Yang, Diandian Deng, Xiaowen Deng, Zhao Chen, Shouzhi Pu

**Affiliations:** 1 Jiangxi Key Laboratory of Organic Chemistry, Jiangxi Science and Technology Normal University, Nanchang 330013, China; 15797638945@163.com (Y.Y.); dengdiandian123456@163.com (D.D.); dengxiaowen3192@163.com (X.D.); 2 Department of Ecology and Environment, Yuzhang Normal University, Nanchang 330103, China

**Keywords:** triphenylamine, carbazole, tetraphenylethylene, mechanochromic, aggregation-induced emission

## Abstract

The development of mechanochromic fluorophors with high-brightness, solid-state fluorescence is very significant and challenging. Herein, highly solid-state emissive triphenylamine, carbazole and tetraphenylethylene-functionalized benzothiadiazole derivatives were developed. These compounds showed remarkable aggregation-induced emission and solvatochromic fluorescence characteristics. Furthermore, these fluorogenic compounds also displayed different mechanically triggering fluorescence responses.

## 1. Introduction

Organic materials with high-brightness, solid-state emission characteristics have attracted much attention due to their application prospects in mechanical sensors, deformation detectors, security systems, memory devices, data storage, fluorescent probes, and various optoelectronic devices [1,2,3,4,5,6,7,8,9,10,11]. In the past decade, smart materials have aroused extensive research enthusiasm in the fields of mechanical sensors, organic light-emitting diodes, and optical storage [12,13,14,15,16,17,18]. In recent years, several strategies have been used to dynamically modulate fluorescence emissions [19,20,21,22]. The Tian group achieved color switchable fluorescence emissions from a single fluorophore in different assembly states [23]. Fluorescence emissions can also be modulated by external stimuli. The type of stimulation can be divided into photochromic, electrochromic, thermochromic, mechanochromic, and so on [24,25,26,27,28,29,30,31,32,33,34,35]. The emission properties of mechanochromic fluorescence materials can be controlled by physical channels, such as anisotropic grinding, shearing, or rubbing [36,37,38,39,40]. These approaches only change molecular stacking patterns or conformations, but do not alter the native chemical structures. However, it is difficult to develop and apply conventional fluorophores due to the aggregation caused by quenching (ACQ). Recently, aggregation-induced emission (AIE) and aggregation-induced emission enhancement (AIEE) have been proposed as fundamental solutions to tackle the challenge of ACQ [41,42]. These approaches open a new path in the creation of robust, solid-state luminescent materials. According to the restriction of intramolecular motions (RIM) mechanism, the steric resistance of aggregated states in luminescent groups with AIE properties interferes with non-radioactive decay channels, allowing the energy of excited states to be dissipated through radiative relaxation [43,44,45]. Thus, luminogenic materials with AIE or AIEE properties show a remarkable emission enhancement when they are aggregated in solids.

Recently, the development of organic stimuli-responsive materials has gained significant attention. Mechanochromic luminescence (MCL) and AIE are promising strategies for the design of novel, robust luminescent materials. On the one hand, benzothiadiazoles are widely used in the construction of fluorescent compounds due to their π-extended structure and electron-withdrawing properties [46,47]. On the other hand, phenanthroimidazoles are rigid, planar, aromatic heterocyclic compounds formed by the fusion of phenanthrene and imidazole rings, with high thermal stability, excellent charge carrier mobility, wide band gap, and excellent fluorescence quantum yields [48,49,50,51]. Simple functional modifications at the N1 and C2 positions of the imidazole ring and the presence of two nitrogen atoms make them bipolar, making phenanthromizole derivatives suitable for use as mechanochromic materials [52].

**Scheme 1:**
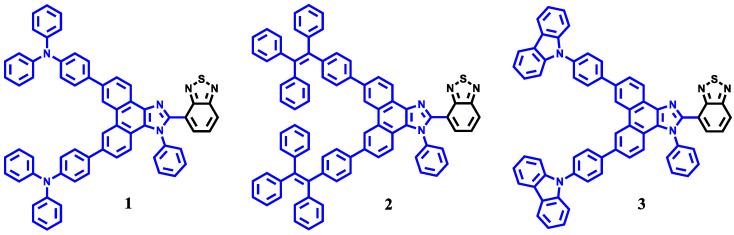
The molecular structures of compounds **1–3**.

Herein, we describe the synthesis of benzothiadiazoles **1**–**3** incorporated with triphenylamine, carbazole, tetraphenylethylene-functionalized phenanthroimidazole unit. These compounds showed solvatochromic fluorescence characteristics and diverse aggregate emission (Scheme 1). More specifically, compound **1** exhibited excellent AIE behavior, compound **2** exhibited remarkable AIEE behavior, and compound **3** exhibited rare aggregation-induced fluorescence discoloration behavior. Furthermore, fluorogenic compounds **1** and **3** also displayed reversible mechanically triggering fluorescence responses.

## 2. Results and Discussion

### 2.1. Aggregation-Inducing Characteristics of Compounds **1**–**3**

After obtaining compounds **1**–**3**, a preliminary study of their aggregation-inducing properties was carried out. The UV-vis absorption and fluorescence (FL) spectra were measured in DMF-H_2_O mixtures with different volume fractions of water (*f*_w_). Absorption bands around 350 nm were assigned to the π-π* and n-π* transitions of the conjugated aromatic skeleton, while the peaks at 400–450 nm were attributed to the intramolecular charge transfer (ICT) from the donor to acceptor (Figure S1). With the increase of water content (*f*_w_), leveled-off tails appeared in the long wave band. This demonstrated the formation of nano-aggregates [53,54].

**Figure 1:**
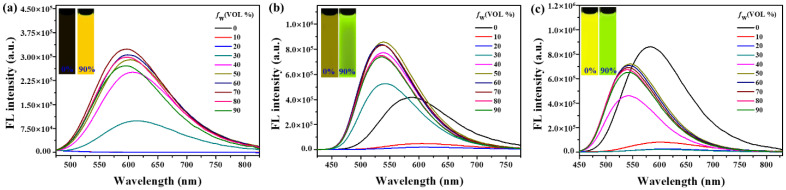
Fluorescence spectra of the dilute solutions of luminogens **1–3** (**a**–**c**) in DMF-H_2_O mixtures with various water contents. Inset: fluorescence images (*f*_w_; excited wavelength: 365 nm, 20 mm).

The drastic differences in AIE properties of compounds **1**–**3** are very interesting. As shown in Figure 1a, Compound **1** showed almost no fluorescence when its diluted DMF solution was excited by light. The most likely explanation for this phenomenon is that the rotational motions of molecular rotors consumed exciton energy and increased nonradiative decay rates, resulting in non-emission [55]. The intensity of the fluorescence emission at around 600 nm increased gradually upon increasing the fraction of water. The maximum PL intensity was observed at 70% water content upon aggregation. As depicted in Figure 1, a dramatic increase of emission intensity revealed the AIE properties of compound **1**.

In addition, the DMF solution of **2** emitted a weak luminescence, and when a little water (<20 vol%) was added to the solution, the fluorescence was further quenched, which can be attributed to the polarity change of the solvent (Figure 1b). The twisted Intramolecular charge transfer (TICT) process may have reduced the emission intensity in this mixed polar solvent. When the water content was increased to above 30 vol%, remarkable fluorescence was observed at about 539 nm, the intensity of which was further increased upon repeated addition of water, demonstrating typical AIEE characteristics.

The most interesting finding, however, is that compound **3** showed a fairly rare changing aggregate fluorescence behavior (Figure 1c). In pure DMF, the emission spectrum of luminogen **3** had a peak at 583 nm and emitted orange fluorescence under UV irradiation. With increasing water content in the DMF solution (<30 vol%), compound **3** displayed a red shift of 24 nm and the emission was quenched gradually. The emission intensity increased gradually as the water fraction increased from 30 to 40%. Then, a sharp intensity increase was noted as the water fraction went up from 40% to 50% and blue shift occurred. With higher water fractions (=90 vol%), the emission was characterized by blue-shift with a maximum at 541 nm.

**Figure 2:**
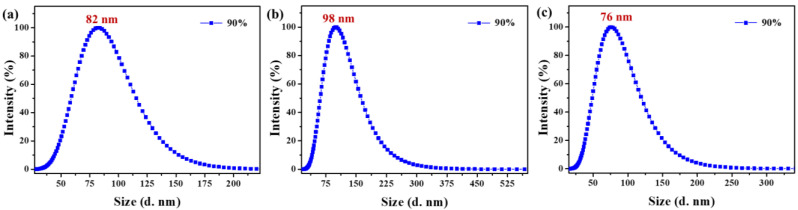
Size distribution curves of **1–3** (**a**–**c**) in DMF-H_2_O mixtures with *f*_w_ = 90%. Concentration: 20 μM.

Dynamic light scattering (DLS) experiments clearly revealed the generation of nanoaggregates of luminogens **1**–**3** with average particle sizes of 82 nm, 98 nm, and 76 nm, respectively (Figure 2). The remarkably increased emissions of compounds **1**–**3** from the aggregates may be due to radiative decay activated by restricted rotor motions. Simultaneously, the twisted conformations of triphenylamine, tetraphenylethylene, and carbazole segments could prolong the intermolecular distance and prevent emission quenching by the reduction of intermolecular π-π interactions, resulting in a new form of luminescence in the aggregate. According to the above analysis, the triphenylamine-, tetraphenylethylene-, and carbazole-functionalized luminogens exhibited AIE, AIEE, and changing aggregate fluorescence, respectively.

### 2.2. Solvatochromic Effect of Compounds **1**–**3**

**Figure 3:**
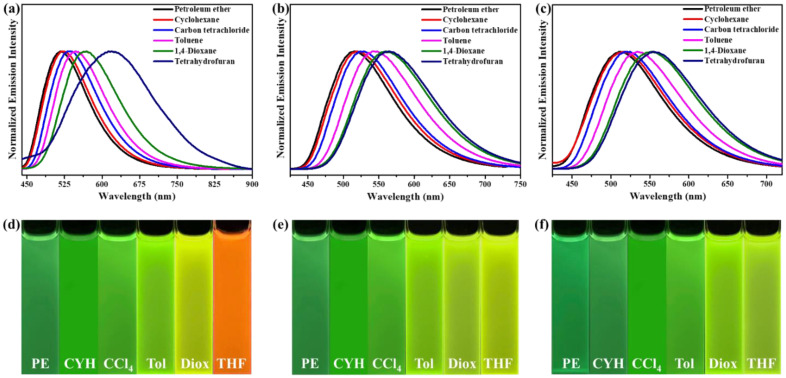
Normalized fluorescence spectra (Excitation wavelength = 365 nm) of compounds **1–3** (**a**–**c**) in different solvents. FL images of compounds **1–3** (**d**–**f**) in different solvents under 365-nm UV illumination. Concentration: 20 μM.

Compounds **1**–**3** possessed typical push-pull substituents, in which the benzothiadiazole unit played the role of electron-acceptor against the electron-donating triphenylamine-, tetraphenylethylene-, and carbazole-functionalized phenanthroimidazole derivatives. The formation of a D-A structure strongly affects the photophysics properties of luminogen in solution, which is strongly dependent on the solvent polarity. Figure 3 shows the fluorescence spectra of compounds **1**–**3** in solvents varying in polarity. Detailed optical data are summarized in Table S1. From non-polar solvents (e.g., petroleum ether) to polar solvents (e.g., tetrahydrofuran), strong solvent-dependent emissions of compound **1**, ranging from 516 nm to 617 nm, were demonstrated, exhibiting a remarkable bathochromic effect. This remarkable solvatochromic phenomenon can be observed in the fluorescence photos. Similarly, with the increase of solvent polarity, compounds **2** and **3** showed the maximum emission wavelength bathochromic shift, i.e., from 515 nm to 564 nm for **2**, and from 510 nm to 554 m for **3**. In a word, these results indicate that the fluorescence behavior of luminogens **1**–**3** was sensitive to solvent polarity, exhibiting strong solvatochromism.

### 2.3. Solid-State Fluorescence Characteristics of Compounds **1**–**3**

**Figure 4:**
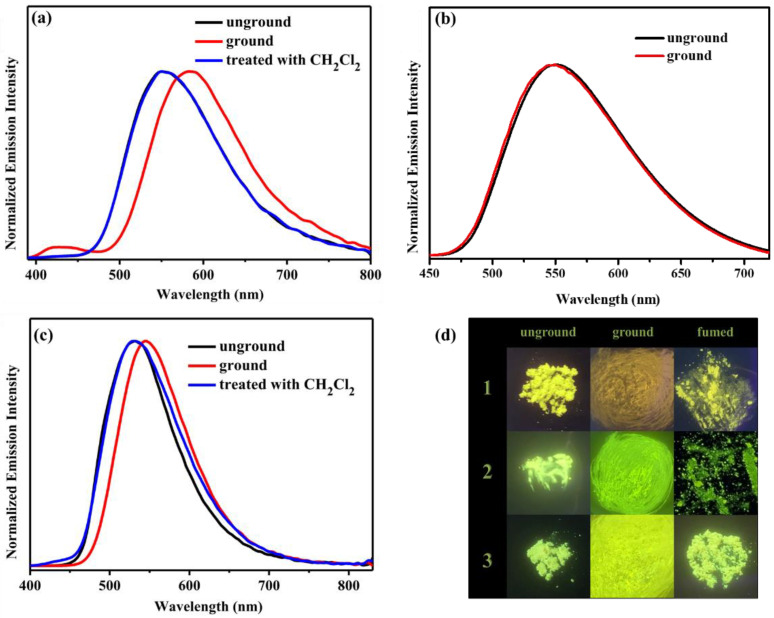
FL spectra of compounds **1** (**a**), **2** (**b**), and **3** (**c**) in various solid states. (**d**) FL photographs of compounds **1–3** in various solid states under 365 nm UV light.

We investigated all compounds in more detail due to their bright luminescence. Upon excitation, compounds **1**–**3** exhibited bright yellowish-green emissions; their emission maxima were determined to be 550 nm, 535 nm, and 532 nm, respectively (Figure 4). We found that solid-state emissions were not regulated by modifying phenanthroimidazole; on the contrary, their mechanochromic properties were changed to a great degree. Specifically, compounds **1** and **3** exhibited excellent reversible mechanochromic behavior, while compound **2** had no mechanochromic properties.

**Figure 5:**
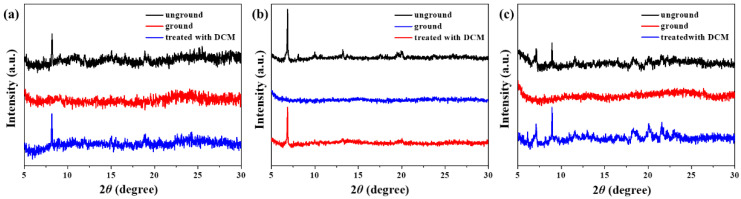
XRD patterns of **1** (**a**), **2** (**b**) and **3** (**c**) in various solid states.

As-prepared sample **1** exhibited an emission peak at 550 nm, with an associated fluorescence quantum yield of 10.12%. However, gentle grinding induced a red-shift of the emission to 583 nm (Φ = 10.35%), which could be ascribed to the amorphous form of the sample (Figure 4a, Table S2). Such an amorphous state was further confirmed by the flat signal in the powder XRD measurement (Figure 5). Meanwhile, the red-shifted emission could be attributed to the collapse of the crystalline lattice by grinding, which resulted in the molecules adopting a more planar conformation [31,56]. Such an amorphous solid can be crystallized by either heating or fuming with an organic solvent vapor. While fuming with the dichloromethane vapor, the amorphous form of **1** was converted into the crystalline form with the emission peak restored to 550 nm. Moreover, this process could be repeated several times, indicating the reversible mechanochromic luminescence property of **1** in response to external stimuli. A similar reversible emission behavior for **3** could be achieved by a repeated grinding/solvent fuming process. Similarly, compound **2** also underwent a process from lattice collapse to reconstruction. However, its maximum fluorescence emission wavelength hardly changed. This suggests that the molecular morphological change from a crystalline to an amorphous state does not necessarily lead to a change in fluorescence.

## 3. Materials and Methods

### 3.1. General Methods

**Scheme 2:**
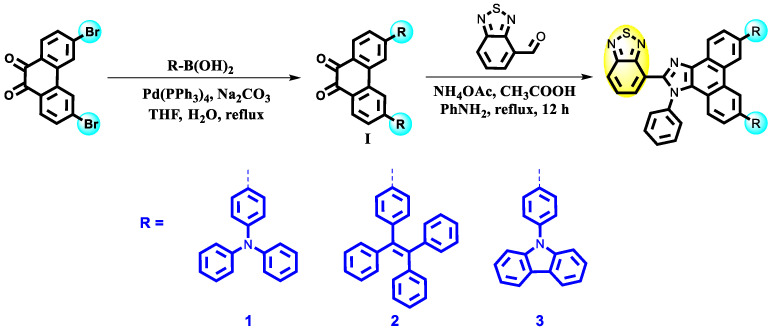
Synthesis of benzothiadiazole-containing dibenzobenzimidazole derivatives **1–3**.

All reactions were performed under an argon atmosphere using standard Schlenk techniques. Unless otherwise stated, all raw materials and solvents were obtained from commercial sources and used without further purification. [4-(1,2,2-triphenylethenyl)phenyl] boronic acid was prepared using published procedure [57]. All of the anhydrous solvents, HPLC grade solvents, and other common organic solvents were purchased from commercial suppliers and used without further purification. The N,N-dimethyl formamide (DMF)/water mixtures with various water fractions were prepared by slowly adding distilled water into the DMF solution of samples. NMR spectra were recorded on a Bruker AV-400 spectrometer at 400 MHz. Coupling constants (*J*) are expressed in hertz (Hz). Chemical shifts (*δ*) of NMR are reported in parts per million (ppm) units relative to internal control (TMS). Mass spectra (MS) were taken in ESI mode on Agilent 1100LCMS (Agilent, Palo Alto, CA, USA). Ultraviolet-visible absorption spectra were measured by an Agilent 8454 UV/Vis spectrophotometer. Fluorescence spectra were recorded on a Hitachi-F-4600 fluorescence spectrophotometer or Edinburgh Instruments FLS 1000 spectrophotometer. Absolute fluorescence quantum yields were measured on an Edinburgh FLS1000 spectrometer. The X-ray diffraction (XRD) patterns of compounds **1**–**3** in different solid states were obtained using a Shimadzu XRD-6000 diffractometer with Ni-filtered and graphite-monochromated Cu Kα radiation (λ = 1.54 Å, 40 kV, 30 mA). Dynamic light scattering (DLS) data were obtained by NanoBrook 90 plus. All reactions were monitored using precoated TLC plates under 254 nm UV light. Silica column chromatography was carried out on silica gel (300–400 mesh). Grinding experiment: The as-prepared solid powders of compounds **1**–**3** were put into a mortar and ground with a pestle at room temperature. Solvent-fuming experiment: the ground samples were suspended above the solvent in a sealed DCM-containing beaker and then exposed to the vapor for 30 s at room temperature. A general synthetic route for compounds **1**-**3** is shown in Scheme 2.

### 3.2. General Synthetic Procedure of Intermediate **I**

To 3,6-Dibromophenanthrene-9,10-dione (3 mmol, 1.0 eq.) were added R-B(OH)_2_ (7.2 mmol, 2.4 eq.) and Pd (PPh_3_)_4_ (0.1 mmol, 3%) in a 100 mL three-necked round-bottom flask. Then, moderate tetrahydrofuran and sodium carbonate solution (20 mmol L^−1^) were added, and the mixture was heated to reflux and stirred for 16 h. After cooling to room temperature, the reaction mixture was extracted with copious DCM (3 × 50 mL), dried (Na_2_SO_4_), and concentrated in vacuo. The combined organic layers were concentrated and subjected to flash column chromatography on silica-gel column.

### 3.3. General Synthetic Procedure and Characterization of Dibenzobenzimidazole Derivatives **1**–**3**

Into a 100 mL three-necked round-bottom were added intermediate **I** (0.3 mmol, 1.0 eq.), 2,1,3-Benzothiadiazole-4-carboxaldehyde (0.3 mmol, 1.0 eq.), aniline (0.45 mmol, 1.5 eq.), ammonium acetate (1.5 mmol, 5 eq.), and acetic acid (30 mL). The mixture was refluxed overnight under an argon atmosphere. After that, the mixture was cooled and filtered, and the solid product was washed in sequence using 15 mL acetic acid/water mixture (volume ratio: 1:1) and 15 mL water. Finally, the crude product was purified by column chromatography.

4,4′-(2-(benzo[c][1,2,5]thiadiazol-4-yl)-1-phenyl-1H-phenanthro [9,10-d]imidazole-6,9-diyl)bis(N,N-diphenylaniline) (**1**): a yellow solid, yield: 78%. ^1^H NMR (400 MHz, CDCl_3_): *δ* 8.99 (s, 1H), 8.71–8.64 (m, 2H), 7.95 (d, *J* = 8 Hz, 1H), 7.84 (d, *J* = 12 Hz, 1H), 7.74–7.67 (m, 4H), 7.52–7.46 (m, 3H), 7.42 (s, 1H), 7.34–7.32 (m, 3H), 7.21–7.17 (m, 7H), 7.14–7.09 (m, 8H), 7.04 (t, *J* = 8, 5H), 6.99–6.94 (m, 6H); ^13^C NMR (100 MHz, CDCl_3_): *δ* 154.8, 153.7, 147.8, 147.8, 147.6, 147.4, 139.3, 138.1, 137.4, 134.8, 133.8, 132.1, 129.5, 129.4, 129.3, 129.0, 128.8, 128.6, 128.2, 127.6, 127.3, 127.1, 124.6, 124.5, 124.4, 123.9, 123.7, 123.6, 123.4, 123.1, 123.1, 122.9, 122.9, 120.3, 118.4; MS-ESI (*m/z*): Found: [M + H]^+^ 915.3206, molecular formula C_63_H_48_N_6_S, requires [M + H]^+^ 915.3270.

4-(1-phenyl-6,9-bis(4-(1,2,2-triphenylvinyl)phenyl)-1H-phenanthro [9,10-d]imidazol-2-yl)benzo[c][1,2,5]thiadiazole (**2**): a yellow solid, yield: 71%. ^1^H NMR (400 MHz, CDCl_3_): *δ* 8.94 (s, 1H), 8.67–8.60 (m, 2H), 7.95 (d, *J* = 8 Hz, 1H), 7.79 (d, *J* = 8 Hz, 1H), 7.68 (d, *J* = 8 Hz, 2H), 7.56 (d, *J* = 8 Hz, 2H), 7.51–7.42 (m, 4H), 7.31–7.26 (m, 5H), 7.17 (s, 1H), 7.06 (s, 8H), 7.03 (t, *J* = 4.0 Hz, 15H), 6.99 (t, *J* = 4.0 6H), 6.96 (s, 2H), 6.92 (d, *J* = 12 Hz, 2H); ^13^C NMR (100 MHz, CDCl_3_): *δ* 154.8, 153.7, 147.7, 143.8, 143.8, 143.7, 143.6, 143.0, 142.9, 141.3, 141.2, 140.6, 140.4, 139.5, 138.6, 138.0, 137.9, 137.8, 137.5, 132.1, 131.7, 131.4, 131.3, 131.3, 129.5, 128.9, 128.7, 128.6, 128.3, 127.7, 127.2, 126.7, 126.6, 126.5, 126.43, 126.4, 125.7, 124.6, 124.6, 124.4, 123.7, 123.6, 123.0, 122.8, 120.7, 119.0; MS-ESI (*m/z*): Found: [M + H]^+^ 1089.3988, molecular formula C_79_H_52_N_4_S, requires [M + H]^+^ 1089.3991.

4-(6,9-bis(4-(9H-carbazol-9-yl)phenyl)-1-phenyl-1H-phenanthro [9,10-d]imidazol-2-yl)benzo[c][1,2,5]thiadiazole (**3**): a yellow solid, yield: 73%. ^1^H NMR (400 MHz, CDCl_3_): *δ* 9.17 (s, 1H), 8.87 (d, *J* = 8 Hz, 1H), 8.82 (d, *J* = 12 Hz, 1H), 8.10 (d, *J* = 4 Hz, 4H), 8.05 (s, 1H), 7.99 (d, *J* = 8 Hz, 2H), 7.90 (d, *J* = 8 Hz, 1H), 7.75 (d, *J* = 8 Hz, 1H), 7.64 (d, *J* = 8 Hz, 2H), 7.56 (s, 3H), 7.49 (s, 4H), 7.46 (s, 1H), 7.43 (s, 2H), 7.40 (s, 3H), 7.37 (d, *J* = 4 Hz, 5H), 7.24 (t, *J* = 8 Hz, 5H), 7.03 (t, *J* = 8 Hz, 1H); ^13^C NMR (100 MHz, CDCl_3_): *δ* 154.8, 153.7, 141.0, 140.9, 139.9, 139.1, 138.1, 137.4, 137.2, 132.2, 129.7, 129.1, 128.9, 128.8, 128.0, 127.6, 127.3, 127.3, 126.0, 125.0, 124.8, 124.3, 124.1, 123.9, 123.5, 123.4, 123.1, 121.1, 120.4, 120.3, 120.1, 120.0, 119.4, 109.9, 109.8. Found: [M + H]^+^ 911.2906, molecular formula C_63_H_38_N_6_S, requires [M + H]^+^ 911.2957.

## 4. Conclusions

In summary, novel benzothiadiazole-based triphenylamine-, carbazole-, and tetraphenylethylene-containing luminogens were designed and synthesized to investigate their AIE, solvatochromic, and mechanofluorochromic characteristics. Luminogen **1** showed a typical AIE effect, luminogen **2** displayed excellent AIEE properties, and luminogen **3** exhibited unconventional changing aggregate fluorescence behavior. As expected, all three compounds also revealed a remarkable solvatochromic effect. In addition, compounds **1** and **3** exhibited reversible mechanofluorochromism phenomena. The PXRD results indicated that the reversible conversion from a crystalline to an amorphous state was responsible for the reversible mechanofluorochromic characteristics of these compounds. This work will be beneficial for the development of mechanical-force sensors with AIE features.

## Data Availability

The data presented in this study are available in Supplementary Materials.

## Supplementary Materials

The following are available online at https://www.mdpi.com/article/10.3390/molecules27154740/s1. Figure S1. UV-Vis absorption spectra of compounds **1–3**. Table S1. Photophysical properties of compounds **1–3**. Table S2. Emission maxima of original, ground and fumed compounds **1–3**. Figures S2–S4. ^1^H NMR spectrum of **1–3** in CDCl_3_. Figures S5–S7. ^13^C NMR spectrum of **1**–**3** in CDCl_3_. Figures S8–S10 Mass spectra of luminogens **1–3**.

## Sample Availability

Not available.
