# Supplementary material for: Triphenylamine, Carbazole or Tetraphenylethylene-Functionalized Benzothiadiazole Derivatives: Aggregation-Induced Emission (AIE), Solvatochromic and Different Mechanoresponsive Fluorescence Characteristics
Source: Molecules. 2022 Jul 25;27(15):4740. doi: 10.3390/molecules27154740 (PMC9331885; doi:10.3390/molecules27154740)
Supplement: Supplementary file 1 [file molecules-27-04740-s001.zip › molecules-1813315-supplementary.pdf]

Electronic Supplementary Information (ESI)

Triphenylamine, carbazole or tetraphenylethylene-functionalized benzothiadiazole derivatives: aggregation-induced emission (AIE), solvatochromic and different mechanoresponsive fluorescence characteristics

Yue Yang [1,‡] Dian-dian Deng [1,‡] Xiao-wen Deng [1], Zhao Chen [1,*] and Shouzhi Pu [1,2,*]

[1] Jiangxi Key Laboratory of Organic Chemistry, Jiangxi Science and Technology Normal University, Nanchang, Jiangxi 330013, PR China; 15797638945@163.com (Y.Y.); dengdiandian123456@163.com (D.-d.D.); 1592958904@qq.com (X.-w.D.); chenzhao666@126.com (Z.C.); pushouzhi@tsinghua.org.cn (S.P.)

[2] Department of Ecology and Environment, Yuzhang Normal University, Nanchang, Jiangxi 330103, PR China

* Correspondence: chenzhao666@126.com (Z.C.); pushouzhi@tsinghua.org.cn (S.P.)

‡ These authors contributed equally to this work.

Table of Contents

1. Figure S1...S2

2. Table S1....……………..S2

3. Table S2....………………..…..S2

4. ¹H and ¹³C NMR spectra of 1-3 in $CDCl_3$.......................................S3

5. Mass spectra of luminogens 1-3…………………………………....S6

1. Figure S1

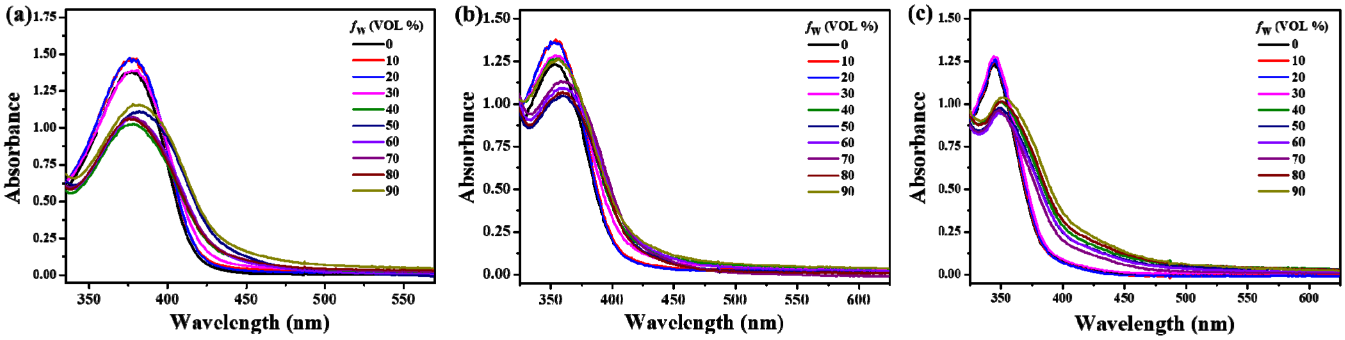

Figure S1. UV-Vis absorption spectra of compounds **1-3** (a-c) in DMF-H_2O mixtures (concentration: 2×10^{-5} mol L^{-1}) with different water fractions (0-90%) at room temperature.

2. Table S1 Photophysical properties of compounds 1-3

Luminogens	λ_{max} nm (PL) [a]					
	PE [b]	CYH [b]	CCl_4 [b]	Tol [b]	Diox [b]	THF [b]
**1**	516	519	531	546	569	617
**2**	515	518	528	541	558	564
**3**	510	515	519	533	551	554

[a] λ_{max} (PL) was measured at exc. = 365 nm and concentration = 1×10^{-5} mol L^{-1}.
[b] PE: Petroleum ether; CYH: Cyclohexane; CCl_4: Carbon tetrachloride; Tol: Toluene; Diox: dioxane; THF: tetrahydrofuran.

3. Table S2 Emission maxima (λ, in nm) of original, ground and fumed compounds 1-3

luminogens	Original (nm)		Ground (nm)		$\triangle\lambda_{em}$ [b] (nm)
	λ_{em} (nm)	Φ Solid	λ_{em} (nm)	Φ Solid	
**1**	**550**	**10.12%**	**583**	**10.35%**	**33**
**2**	**535**	**26.25%**	**534**	**26.87%**	**-**
**3**	**532**	**20.63%**	**545**	**23.98%**	**13**

[a] Absolute fluorescence quantum yield determined by calibrated integrating sphere system at 298 K under air.

[b] $\triangle\lambda_{em} = |\lambda_{Grinding} - \lambda_{Original}|$.

4. ^{1}H and ^{13}C NMR spectra of 1-3 in $CDCl_3$

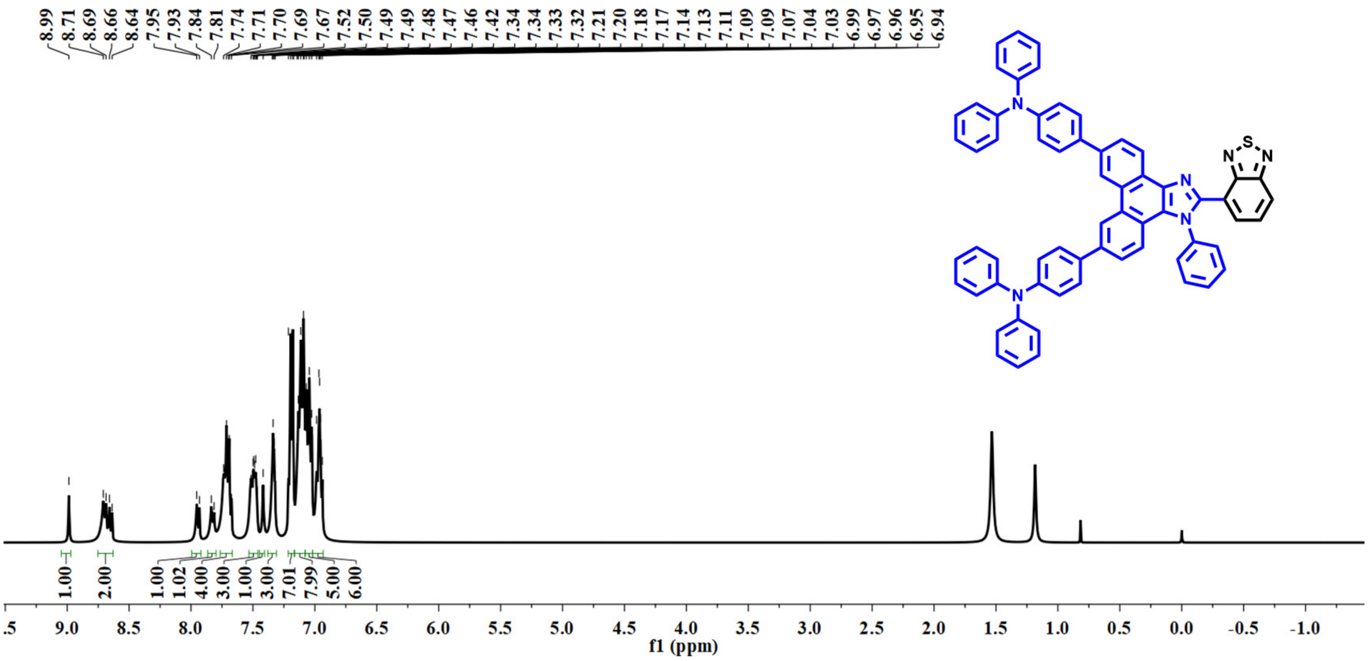

Figure S2 ^{1}H NMR spectrum of **1** in $CDCl_3$.

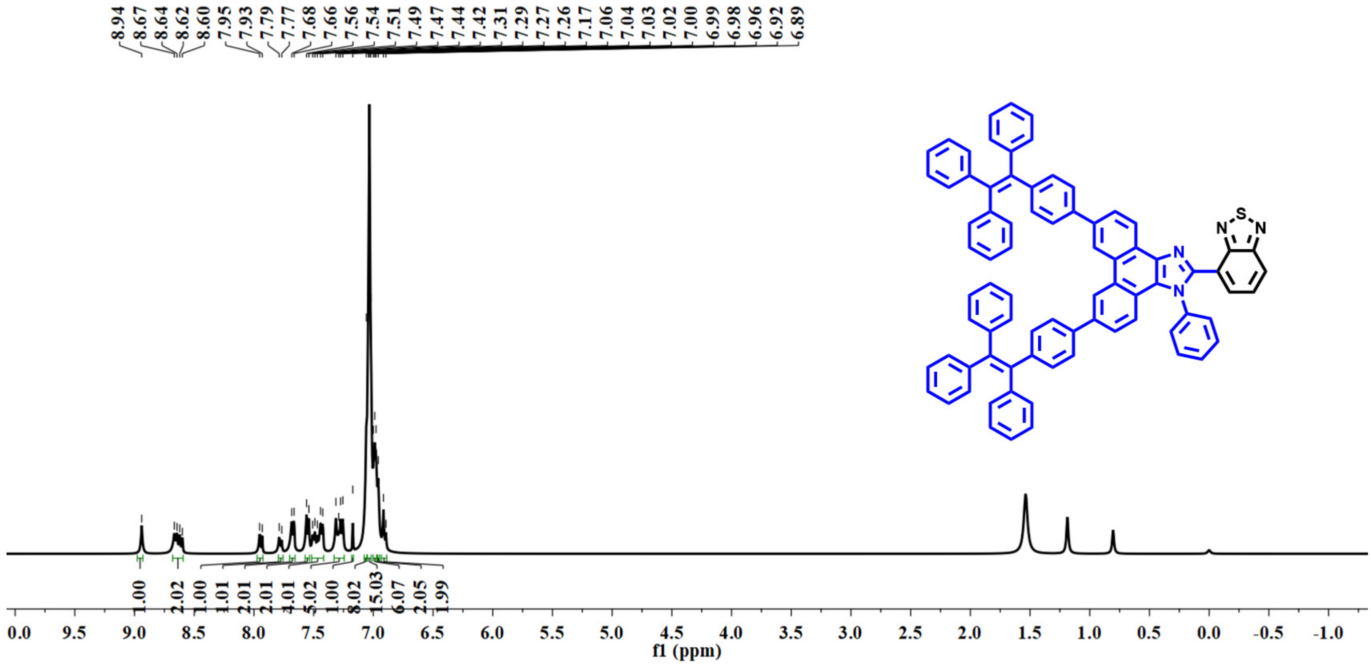

Figure S3 ^{1}H NMR spectrum of **2** in $CDCl_3$.

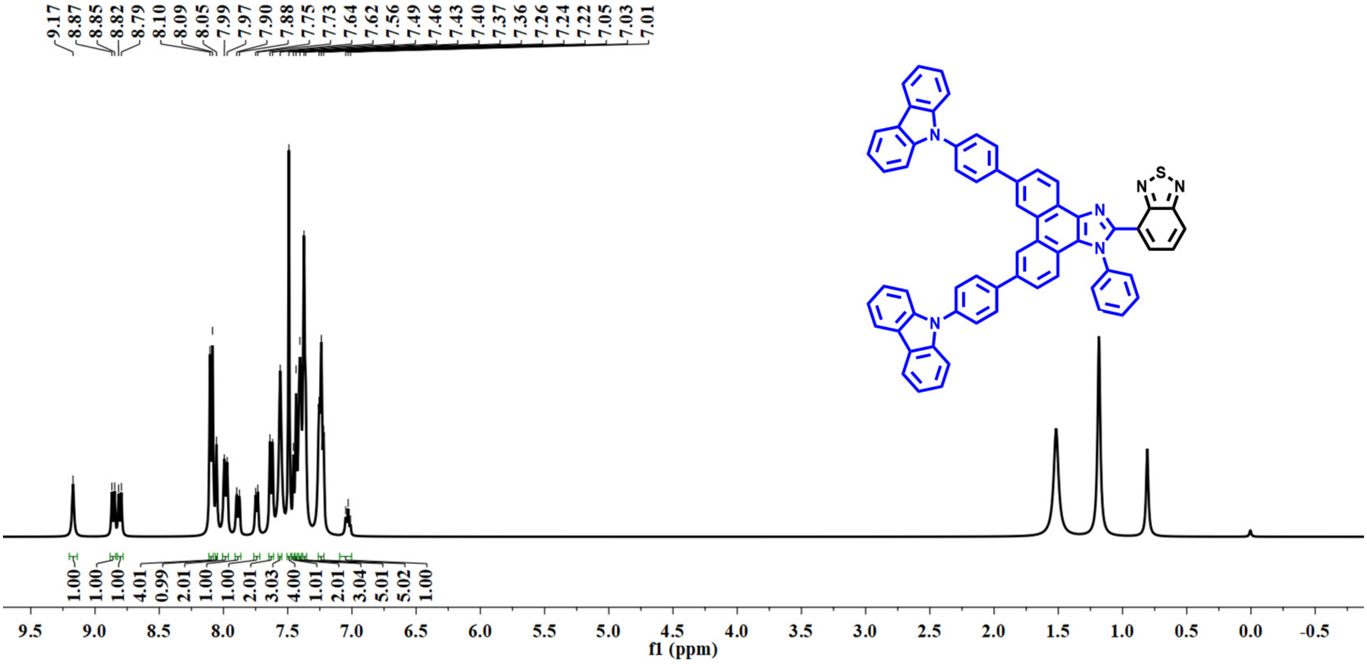

Figure S4 ^{1}H NMR spectrum of **3** in $CDCl_3$.

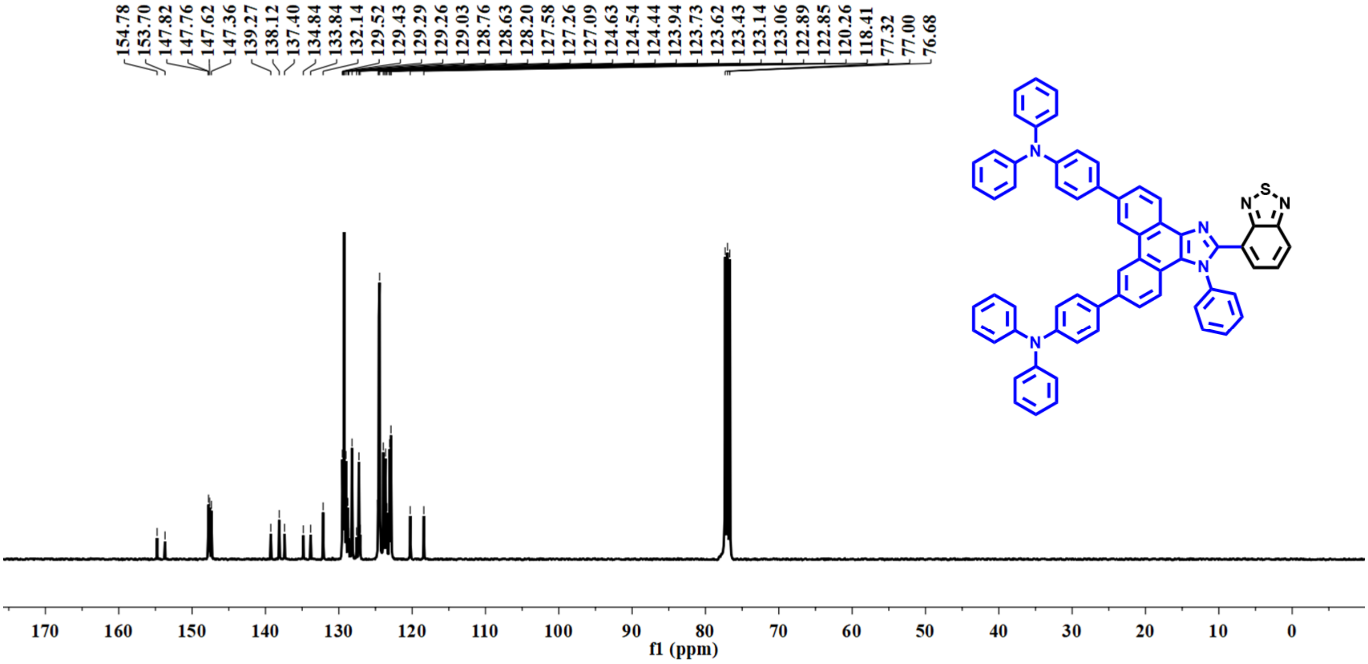

Figure S5 ^{13}C NMR spectrum of **1** in $CDCl_3$.

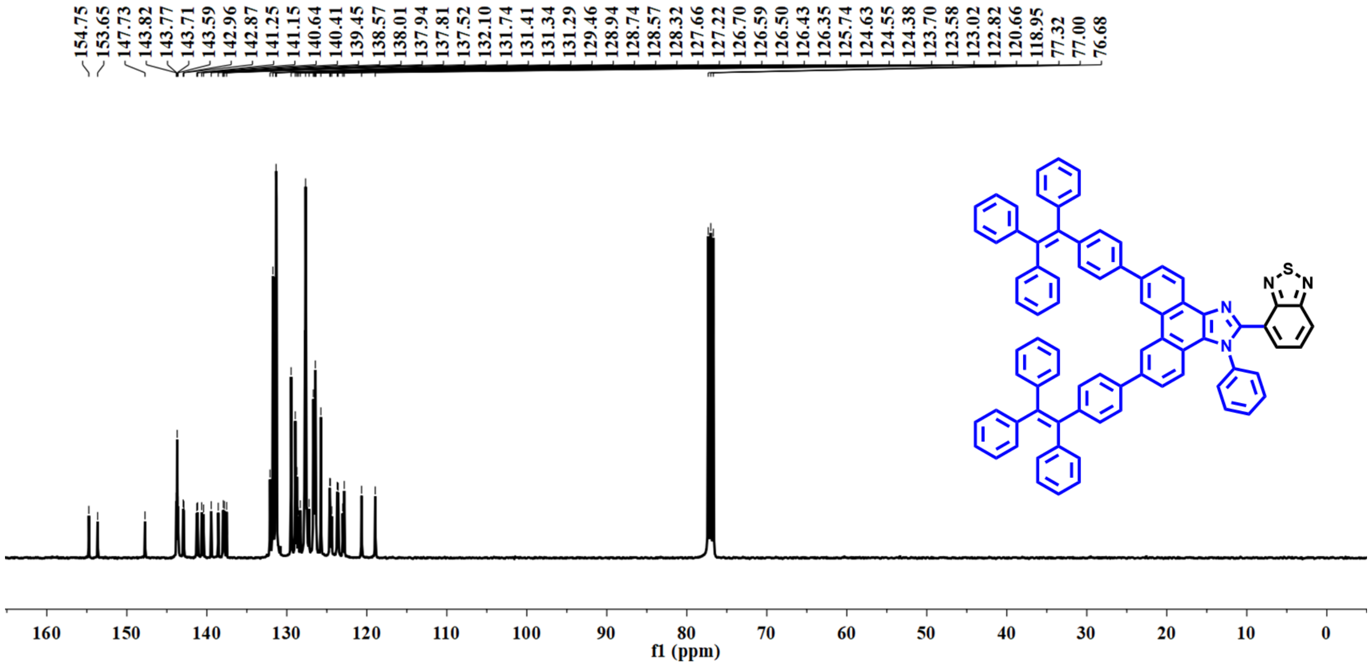

Figure S6 ^{13}C NMR spectrum of **2** in $CDCl_3$.

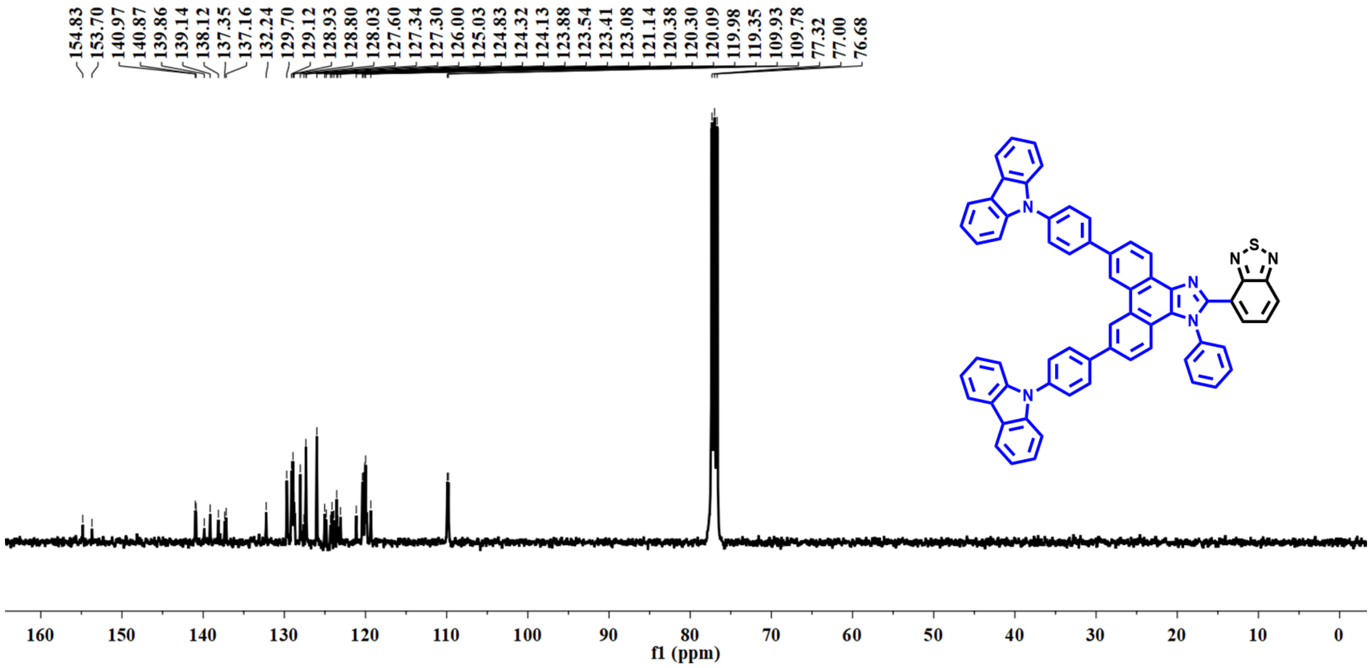

Figure S7 ^{13}C NMR spectrum of **3** in $CDCl_3$.

4. Mass spectra of luminogens 1-3

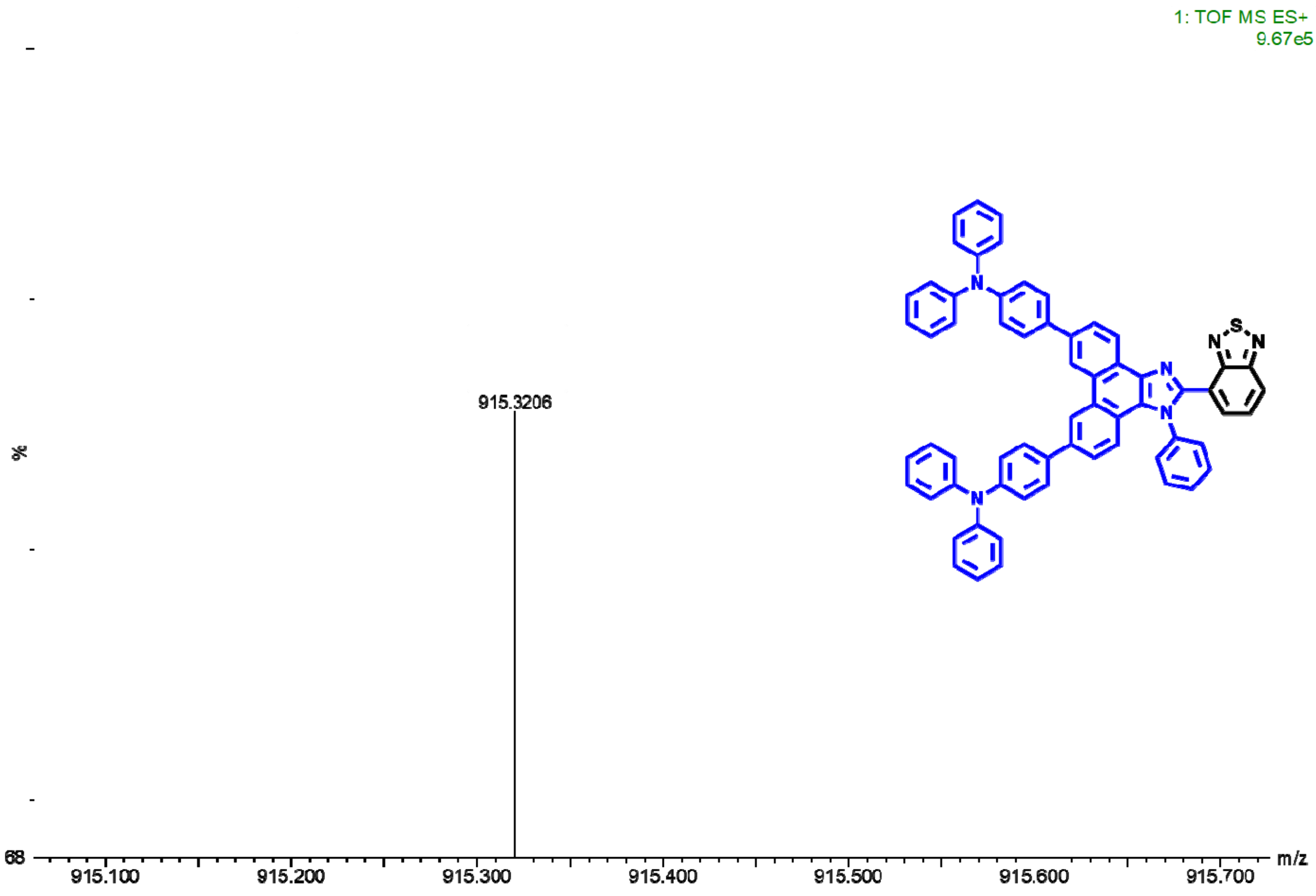

Figure S8 Mass spectra of luminogen 1

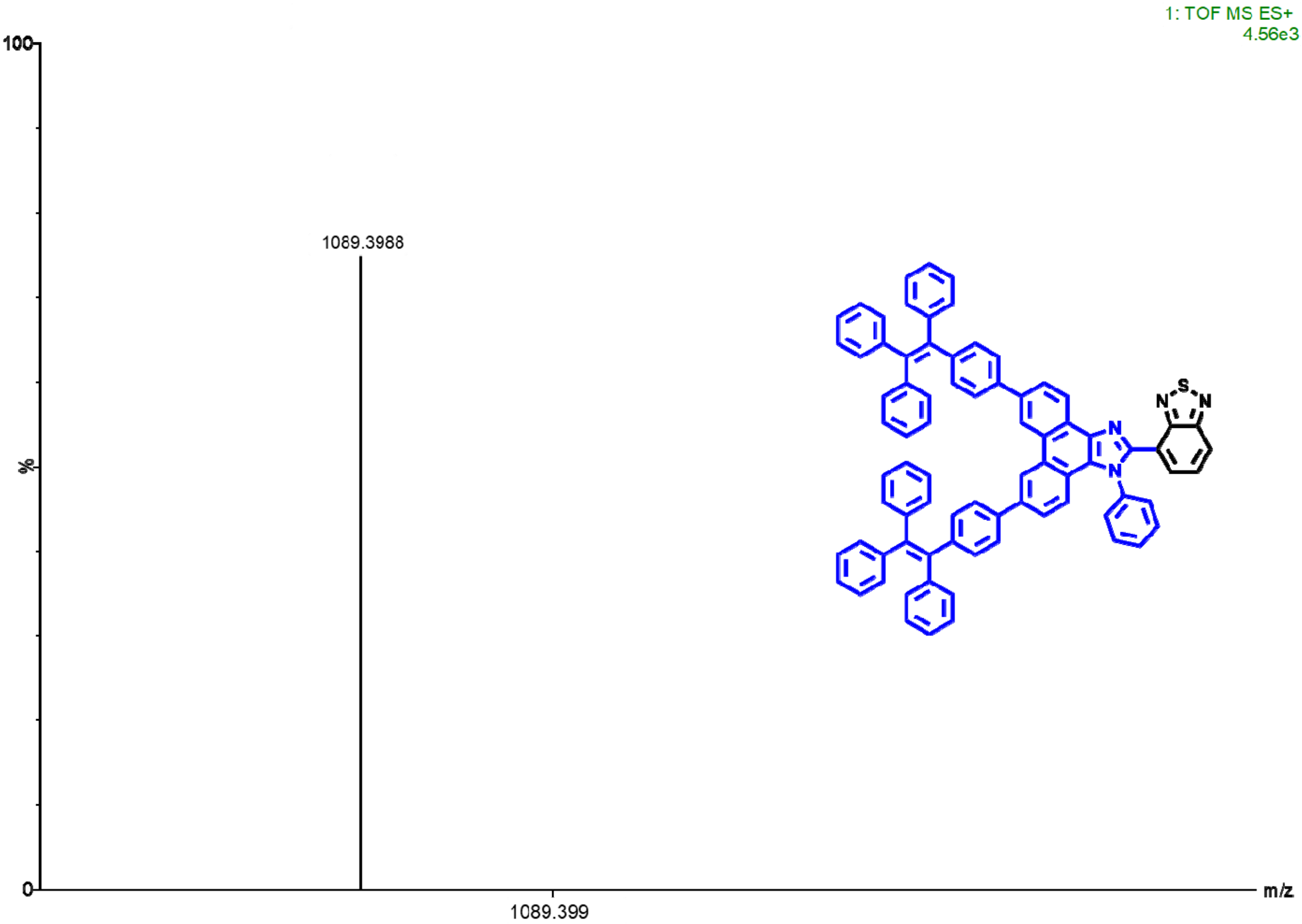

Figure S9 Mass spectra of luminogen 2

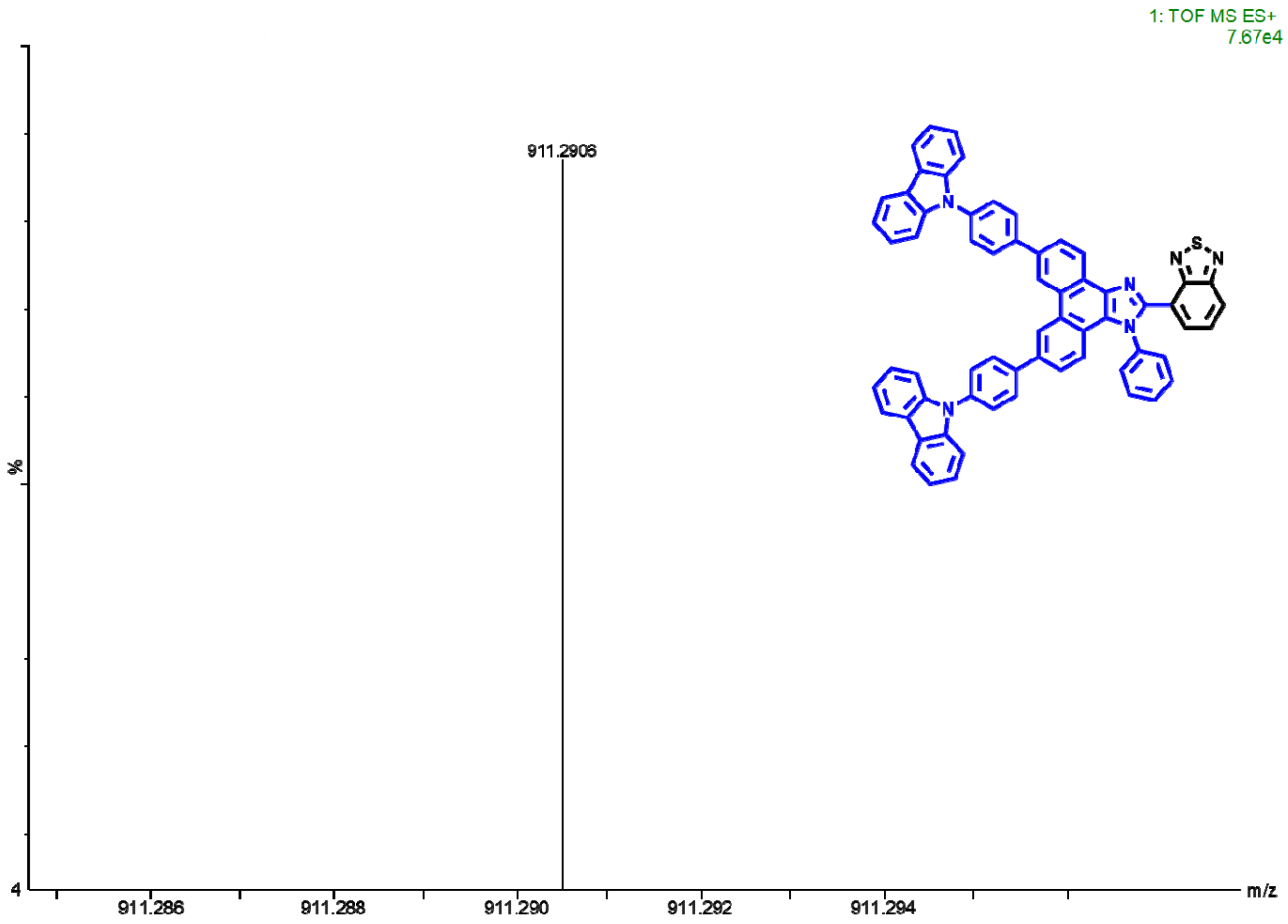

Figure S10 Mass spectra of luminogen 3
